# Structural and functional brain alterations in anorexia nervosa:A multimodal meta‐analysis of neuroimaging studies

**DOI:** 10.1002/hbm.25602

**Published:** 2021-07-23

**Authors:** Ting Su, Jiaying Gong, Guixian Tang, Shaojuan Qiu, Pan Chen, Guanmao Chen, Junjing Wang, Li Huang, Ying Wang

**Affiliations:** ^1^ Medical Imaging Center First Affiliated Hospital of Jinan University Guangzhou China; ^2^ Institute of Molecular and Functional Imaging Jinan University Guangzhou China; ^3^ Department of Radiology Six Affiliated Hospital of Sun Yat‐sen University Guangzhou China; ^4^ Department of Applied Psychology Guangdong University of Foreign Studies Guangzhou China

**Keywords:** anorexia nervosa, meta‐analysis, multimodal, resting‐state functional imaging, voxel‐based morphometry

## Abstract

Anorexia nervosa (AN) is a complex psychiatric disorder with poorly understood etiology. Numerous voxel‐based morphometry (VBM) and resting‐state functional imaging studies have provided strong evidence of abnormal brain structure and intrinsic and functional activities in AN, but with inconsistent conclusions. Herein, a whole‐brain meta‐analysis was conducted on VBM (660 patients with AN, and 740 controls) and resting‐state functional imaging (425 patients with AN, and 461 controls) studies that measured differences in the gray matter volume (GMV) and intrinsic functional activity between patients with AN and healthy controls (HCs). Overall, patients with AN displayed decreased GMV in the bilateral median cingulate cortex (extending to the bilateral anterior and posterior cingulate cortex), and left middle occipital gyrus (extending to the left inferior parietal lobe). In resting‐state functional imaging studies, patients with AN displayed decreased resting‐state functional activity in the bilateral anterior cingulate cortex and bilateral median cingulate cortex, and increased resting‐state functional activity in the right parahippocampal gyrus. This multimodal meta‐analysis identified reductions of gray matter and functional activity in the anterior and median cingulate in patients with AN, which contributes to further understanding of the pathophysiology of AN.

## Graphical abstract

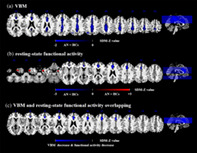

## 1 INTRODUCTION

Anorexia nervosa (AN) is a psychiatric disorder characterized by severe restriction of energy intake leading to significantly low body weight, intense fear of gaining weight or persistent behavior that interferes with weight gain, and distortion in the perception of one's body weight or shape (Washington, 2013). It is an important cause of physical and psychosocial morbidity in adolescent girls and young adult women, but is much less frequent in men (Fairburn & Harrison, 2003). AN is a disorder with relatively high rates of morbidity and mortality, as the lifetime prevalence of AN (diagnosed by fifth edition of The Diagnostic and Statistical Manual of Mental Disorders [DSM‐5]) among women might be up to 4% (Smink, van Hoeken, & Hoek, 2013; Washington, 2013), and the crude rate of mortality due to all causes for AN was up to 5.9% (Sullivan, 1995). No body system is spared from the adverse sequelae of AN (Westmoreland, Krantz, & Mehler, 2016), and the long‐term prognosis is relatively poor (Steinhausen, 2002). However, effective treatments for AN remain limited (Zhang et al., 2013; Zipfel, Giel, Bulik, Hay, & Schmidt, 2015), and the neural underpinnings of this disease are not well understood.

Multi‐modal neuroimaging has been recently applied to investigate the structural and functional abnormalities of the brain in AN (Cha et al., 2016; Gaudio, Wiemerslage, Brooks, & Schioth, 2016). Several meta‐analyses have indicated brain structural alterations in AN. A meta‐analysis and qualitative review of morphological changes in the brain showed reduced gray matter volume (GMV) in the hippocampal, cingulate, midbrain, cerebellar regions, and the lateral occipital cortex, and increased GM in the dorsolateral prefrontal cortex and medial orbitofrontal cortex in AN (Seitz et al., 2014). Another meta‐analysis of voxel‐based morphometry (VBM) study (228 patients with AN; 240 controls) demonstrated decreased GMVs of the left hypothalamus, left inferior parietal lobe, right lentiform nucleus, and the right caudate, suggesting that excessive restrained eating as found in patients with AN, correlates with structural changes in the brain analogous to clinical symptoms (Titova, Hjorth, Schioth, & Brooks, 2013). Zhang et al. studied 389 patients with AN and 410 controls, and identified decreased GMV in the bilateral median and posterior cingulate cortices extending to the bilateral precuneus, and the supplementary motor area in the AN patients (Zhang et al., 2018). Given that some VBM studies were not included in the previous meta‐analyses and several VBM studies have been conducted in recent years (Lazaro et al., 2013; Mainz, Schulte‐Ruther, Fink, Herpertz‐Dahlmann, & Konrad, 2012; Muhlau et al., 2007; Nickel et al., 2017; Oliva et al., 2020; Seitz et al., 2015; Wagner et al., 2006), an updated meta‐analysis is needed to extend and/or modify previous structural findings in AN.

Functional magnetic resonance imaging (fMRI) has been used to identify abnormalities of regional brain function evoked by specific tasks or in the resting state. In contrast to task‐based fMRI studies, resting‐state functional magnetic resonance imaging (rs‐fMRI) removes some performance‐related confounders, and therefore could measure task‐independent changes in brain function in humans (Guerra‐Carrillo, Mackey, & Bunge, 2014). Meta‐analyses of resting‐state functional imaging studies have been conducted in psychiatric disorders such as major depressive disorder, attention‐deficit/hyperactivity disorder, and autism spectrum disorder, obsessive–compulsive disorder, internet gaming disorder (Gray, Muller, Eickhoff, & Fox, 2020; Lukito et al., 2020; Norman et al., 2016; Yao et al., 2017), and so forth. However, there is no meta‐analysis of resting‐state functional imaging studies on AN. Resting‐state imaging studies including regional homogeneity (ReHo) (Seidel et al., 2019; Yue, 2013), (fractional) amplitude of low frequency fluctuations (fALFF/ALFF) (Cicerale et al., 2013; Lai et al., 2020; Seidel et al., 2019), together with brain blood flow studies using arterial spin labeling (ASL) (Sheng, Lu, Liu, Thomas, & McAdams, 2015; Wierenga et al., 2017), positron emission tomography (PET) (Gordon et al., 2001; Pasanisi et al., 2013; Zhang et al., 2013) and single‐photon emission computed tomography (SPECT) (Jinjue et al., 2007; Kojima et al., 2005; Naruo et al., 2001; Takano et al., 2001; Yonezawa, Otagaki, Miyake, Okamoto, & Yamawaki, 2008) have contributed to understanding the regional brain function in AN. However, these studies have shown relatively inconsistent results for AN, such as abnormal neural activity in the frontal lobe, hippocampus/parahippocampus (Seidel et al., 2019; Takano et al., 2001; Yue, 2013), parietal lobe (Kojima et al., 2005; Zhang et al., 2013), cingulate cortex (Cicerale et al., 2013; Kojima et al., 2005; Naruo et al., 2001; Wierenga et al., 2017), subcallosal gyrus (Yonezawa et al., 2008; Zhang et al., 2013), and so forth. The reasons for the inconsistent or conflicting findings, may include small sample sizes, age ranges, clinical symptoms, medical comorbidity, duration of illness, and the technical methods of data acquisition and analysis. Therefore, it is important to conduct a meta‐analysis to identify convergent abnormal regional function of AN in the resting‐state functional imaging studies.

Therefore, a multimodal coordinate‐based meta‐analysis of VBM and resting‐state functional studies was conducted to investigate the structural and functional alterations in the brain in AN, and to explore whether there is a structural basis for the functional impairment in specific brain regions of AN. Based on the previous empirical studies investigating VBM and resting‐state functional activity in AN, we speculated that structural and functional changes in AN would be primarily located in the cingulate cortex, and parietal frontal lobe. Furthermore, the present study used Seed‐based d Mapping with Permutation of Subject Images (SDM‐PSI), a software with stricter correction than Anisotropic effect‐size version of Seed‐based d Mapping (AES‐SDM), to explore localized changes of neural function and GMV between AN patients and controls. SDM‐PSI is a novel method with a less biased estimation of the population effect size and the use of random‐effects models and threshold‐free cluster enhancement (TFCE) statistics, which identifies brain regions consistently altered by testing for spatial convergence across findings from published neuroimaging studies (Samartsidis, Montagna, Nichols, & Johnson, 2017; Tench, Tanasescu, Constantinescu, Cottam, & Auer, 2020). The present study offered a more precise understanding of the pathophysiology of AN, and provided potential targets for neuromodulation and other interventions.

## 2 METHODS AND MATERIALS

### 2.1 Data source

A comprehensive and systematic literature search of the following databases: Web of Science, Embase, PubMed, Sinomed, China National Knowledge Infrastructure, and WanFang databases for VBM and resting‐state functional imaging studies published between January 1990 and December 2020, was conducted. We used the following MESH terms and keywords for the search: (1) (“Voxel‐based morphometry” OR “VBM” OR “morphometry”) AND (“eating disorders” OR “anorexia nervosa” OR “anorexia”). (2) (“neuroimag*” OR “fMRI” OR “cerebral blood flow” OR “CBF” OR “positron emission tomography” OR “PET” OR “single photon emission computed tomography” OR “SPECT” OR “arterial spin labeling” OR “ASL” “ALFF” OR “ReHo” OR “resting‐state”) AND (“eating disorders” OR “anorexia nervosa” OR “anorexia”). Besides synonyms of these terms were used, we also manually conducted an additional search within the references lists of the included articles and prior AN‐related meta‐analyses studies for additional qualified studies.

### 2.2 Study selection

Eligible VBM or resting‐state functional imaging studies fulfilled the following inclusion criteria: (a) they compared GMV or resting‐state functional imaging between AN and HCs at the whole brain level; (b) the study was an original article limited to study of human subjects (not as an abstract or a review) in a Chinese or English language journal of peer‐reviewed; (c) articles reporting peak coordinates of significant clusters in standard MNI or Talairach space; (d) When the original manuscripts' details were not reported, it could be available by making a reasonable request to the corresponding author.

**FIGURE 1:**
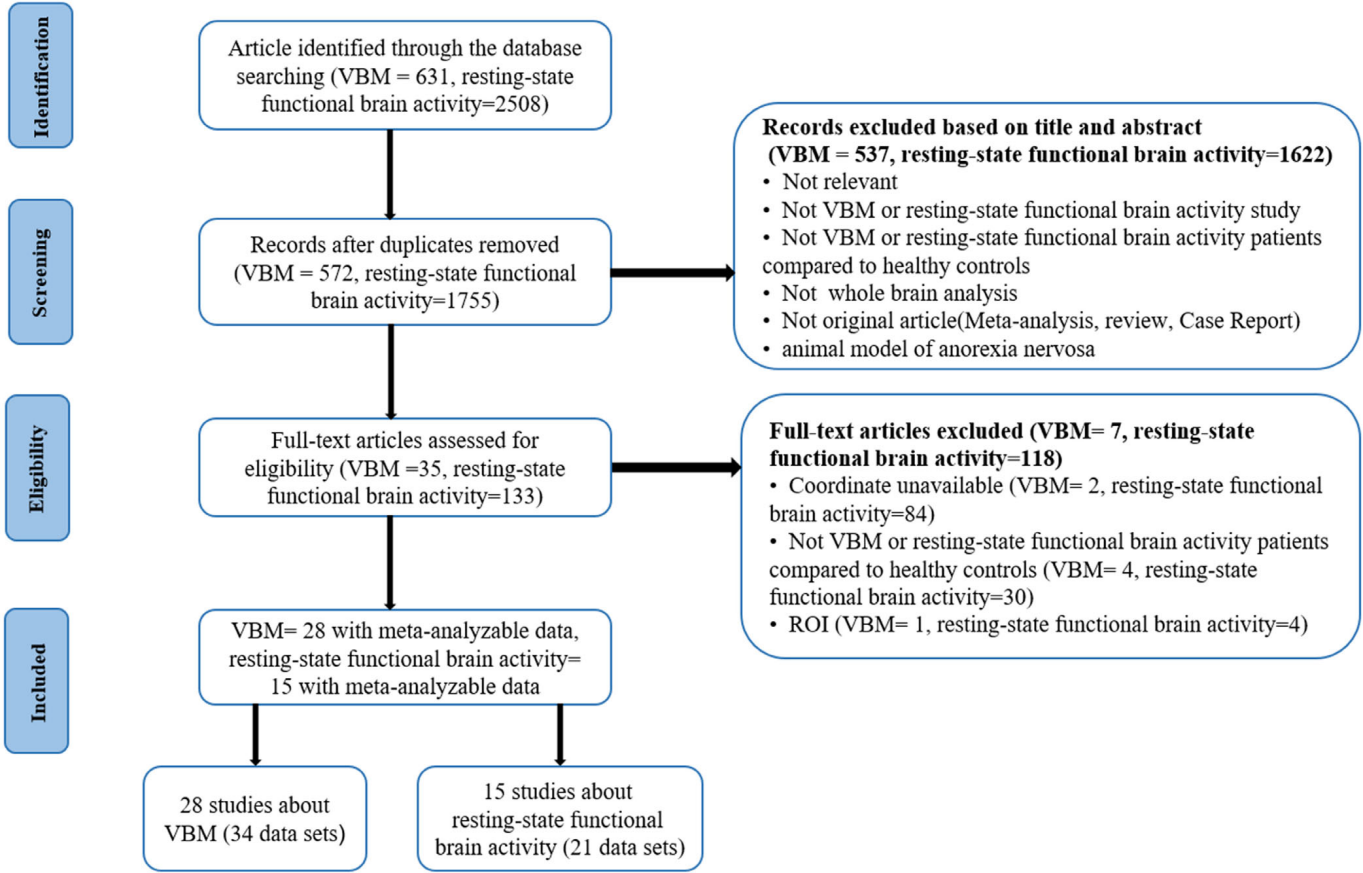
Flow chart of meta‐analysis of voxel‐based morphometry (VBM) and resting‐state functional imaging studies of patients with anorexia nervosa (AN)

Studies were excluded based on the following criteria: (a) analysis focus on specific regions of interest (ROI); (b) sample size was less than three either in the AN group or the HC group; (c) studies reported significant results without listing the three‐dimensional coordinates; (d) the authors did not provide neuroanatomical coordinates after contacting with them by telephone or email; (e) Data were used repeatedly among much paper (Figure 1).

### 2.3 Quality assessment and data extraction

We used a 10‐point checklist to assess individual study quality for meta‐analyses, which focused on the clinical and demographic aspects of the study samples and the imaging methodology (Table S9). This checklist was based on previous meta‐analytic studies (Chen et al., 2015; Gong et al., 2020; Gong et al., 2020). Three investigators (J. Y. G, S. J. Q, and T. S.) searched, evaluated, and selected the retrieved studies, extracted, and cross‐checked data independently following guidelines for neuroimaging meta‐analyses promoted by Müller and colleagues (Muller et al., 2018). For each study, the following data were extracted: the first author's last name, year of publication, demographic information (age and gender), sample size, illness variables (Body Mass Index [BMI], AN type and AN stage (acute vs. recovered), age at onset, illness duration), imaging parameters. The peak coordinates showing differences between subjects and controls were extracted by the SDM method (Radua et al., 2014). Inconsistent information was resolved by a fourth investigator (Y. W.) after comparing the results.

### 2.4 Data analysis

#### 2.4.1 Main meta‐analysis

##### VBM

The whole‐brain VBM meta‐analysis of structural imaging studies was used to determine the structural substrates of altered cerebral volume in AN, which was conducted using SDM‐PSI software package (version 6.21 for Windows) in a standard process (www.sdmproject.com). The SDM‐PSI approach uses effect sizes to combine reported peak coordinates that are extracted from databases with statistical parametric maps, and it imputes the brain maps of the effect size of GMV differences between patients and HCs for each study and then conducts a standard random‐effects meta‐analysis (Dugre et al., 2020; Radua et al., 2012; Sheng et al., 2020). The SDM‐PSI uses standard statistical procedures to control the family wise error rate (FWE) (Albajes‐Eizagirre, Solanes, Vieta, & Radua, 2019). We performed the analysis as described in the SDM‐PSI tutorial and related publications (Lim, Radua, & Rubia, 2014; Radua et al., 2014) and used MRIcron software (www.mricro.com/mricron/) to visualize SDM‐PSI maps.

In order to identify abnormal brain intrinsic and functional activity in AN, the SDM‐PSI approach is briefly described here. The coordinates and height of the peaks (its t‐value or z‐value, but a *p*‐value is also useful) with poorer and greater hemodynamic activity for each study compared with HC were extracted separately. A standard MNI map of the GMV differences was then separately recreated for each data set using an anisotropic Gaussian kernel. The mean map was finally generated by voxel‐wise calculation of the random‐effects mean of the data set maps, weighted by the sample size, intra‐data set variability, and between‐data set heterogeneity. To optimally balance false positives and negatives, we used the default SDM kernel size and thresholds (full anisotropy = 1, isotropic full width at half maximum [FWHM] = 20 mm, and voxel = 2 mm). FWE correction for multiple comparisons using common permutation tests; and use of TFCE in statistical thresholding (*p* < .05 and cluster extent = 10 voxels) (Albajes‐Eizagirre et al., 2019). It should be noted that this FWHM kernel is intended to assign indicators of proximity to reported coordinates but not to smooth any different images. We conducted the meta‐analysis in total patients and the subgroup analysis of AN with no psychiatric comorbidities within VBM studies.

##### Resting‐state functional imaging

Consistent with the meta‐analysis of VBM studies, a similar procedure was performed to select studies related to functional brain activity analysis. We extracted peak coordinates and effect size (e.g., t‐values) of differences in functional brain activity between patients with AN and HCs from each data set. Functional brain activity meta‐analysis was also performed with the above‐mentioned SDM analysis algorithm.

#### 2.4.2 Subgroup meta‐analysis

##### VBM

Subgroup meta‐analysis was conducted when the number of data sets was sufficient (*n* ≥ 10). Analysis would be performed in five subgroups, including (a) adult, (b) female, (c) acute patients with AN, and (d) AN with no psychiatric comorbidities. We did not perform the subgroup analysis of untreated patients because of the small amount of data sets (*n* < 10).

##### Resting‐state functional imaging

Subgroup meta‐analysis was conducted when the number of data sets was sufficient (*n* ≥ 10). Subgroup would be performed in AN with no psychiatric comorbidities. We did not perform the following subgroup analysis because of the small number of data sets (*n* < 10): (a) adult, (b) acute, and (c) untreated patients.

#### 2.4.3 Jackknife sensitivity analysis

To test the repeatability of the results, we performed a jackknife sensitivity analysis, repeating the same analysis K times iteratively (K represents the number of data sets included), leaving out one data set each time (Radua et al., 2014; Radua & Mataix‐Cols, 2009). If the previous major brain regions remain significant in all or most of the combinations of studies, then the findings are considered to be highly repeatable.

#### 2.4.4 Analysis of heterogeneity and publication bias

A heterogeneity analysis was conducted using a random effects model with *I* ^2^ statistics to explore unexplained between‐study variability for every significant cluster with a peak MNI coordinate reported in the main meta‐analysis results. Heterogeneous brain regions were obtained using the default SDM kernel size and thresholds (FWHM = 20 mm, *p* = .005, uncorrected for FDR, peak height Z = 1, cluster extent = 10 voxels) (Lim et al., 2014), with *I* ^2^ < 50% indicating low heterogeneity.

To inspect the publication bias, Egger's tests were performed for quantitative examination and funnel plots were created for visual inspection by extracting the values from significant peaks (Albajes‐Eizagirre et al., 2019). *p* < .05 and an asymmetric plot were considered statistically significant.

#### 2.4.5 Meta‐regression analyses

Meta‐regression analyses were carried out to explore the associations between the analytic results and clinical variables (e.g., illness duration, age, and BMI). The results were weighted by the square root of the sample size. To minimize the reporting of spurious relationship, we selected a more conservative threshold of *p* = .05, TFCE correction, cluster extent = 10 as suggested by the authors of the SDM‐PSI method, requiring abnormalities to be detected both in the slope and in one of the extremes of the regressor, and discarding findings in regions other than those detected in the main analyses.

### 2.5 Multimodal conjunction of abnormal GMV and resting‐state functional activity in AN

To localize brain regions with both GMV and resting‐state functional activity abnormalities in AN, between‐group contrasts of multimodal brain imaging were summarized in a meta‐analytic map (Radua, Romeo, Mataix‐Cols, & Fusar‐Poli, 2013). We identified multimodally affected brain regions by computing the overlapping *p* values of the GMV and resting‐state functional activity. The method we implemented in SDM considered the underlying noise of the meta‐analytic estimation. The voxel‐level threshold was *p* < .0025 because of four tails.

## 3 RESULTS

### 3.1 General overview of studies

We identified 28 included VBM studies with 34 data sets, recruited a total of 660 AN patients (99.3% female; mean age = 22.22 years; illness duration = 37.81 months; mean BMI = 16.61) and 740 HCs (99.4% female; mean age = 22.92 years; mean BMI = 21.42); and 15 resting‐state functional imaging studies reporting 21 data sets comprised 425 AN patients (all are females; mean age = 21.23 years; mean illness duration = 23.74 months; mean BMI = 15.95) and 461 HCs (all are females; mean age = 22.26 years; mean BMI = 20.91). AN and HC groups were mainly composed of female.

We found no significant differences between participants with AN and their HC counterparts with respect to age in VBM studies (*t* = −0.508; *p* = .613) and resting‐state functional imaging studies (*t* = −0.951; *p* = .347). In the VBM studies, the sex distribution of AN patients and HC was matched (*χ2* = 0.047; *p =* 0.828). Tables 1 and 2 are briefly summarized the demographic data, clinical and imaging characteristics of the studies.

**TABLE 1:** Demographic, clinical, and imaging characteristics of the included studies of VBM

Study	Demographic characteristics						Clinical characteristics (AN patients)					Imaging characteristics				
	Participants (female), *n*		Mean age ± *SD*, year		Age group	AN state	Comorbid	Illness duration (months)	Age at onset (years)	BMI		Scanner	Software	FWHM (mm)	Threshold	Quality score^a^
	AN	HC	AN	HC						AN	HC					
(Wagner et al., 2006)^b^	14(14)	31(31)	23.7±5.3	26.8±7.3	Adult	Recovered	NO	28.7±20.4	18.2±4.5	21.2±2.0	21.9±2.0	1.5T MRI	SPM2	12	*p*_FDR_ <.05	10
	16(16)		27.4±7.2		Adult	Recovered	NO	39.5±52.7	15.7±2.7	21.2±1.5	NA					
(Muhlau et al., 2007)	22(22)	37(37)	27.2	27.4	Adult	Recovered	NO	116	17.6	19.8	21.1	1.5 T MRI	SPM2	14	*p*_uncorrected_ <.01	10
(Castro‐Fornieles et al., 2009)	12(11)	9(8)	14.5±1.5	14.6±3.2	Adolescent	NA	NO	NA	NA	14.8±2.0	NA	1.5 T MRI	SPM5, FSL	NA	*p*_FWE_<.05	9
(Suchan et al., 2010)	13(13)	14(14)	26.8±8.4	29.5±8.2	Adult	Chronic	NO	66.0±60.0	NA	16.0±1.3	22.0±2.1	1.5 T MRI	SPM5	12	*p*_FWE_ <.05	9.5
(Boghi et al., 2011)^b^	21(21)	27(27)	29.0±10.0	30.8±8.7	Adult	Recovered/acute	NO	135.6±145.2	NA	15.5±1.8	21.9±1.5	1.0 T MRI	SPM2	12	P_FDR_ <.05	9.5
	10(10)	13(13)	21.4±2.5	22.8±1.5	Adult	Acute	NO	22.8±15.6	NA	15.0±1.2	21.7±1.7	NA		NA	NA	
	11(11)	14(14)	35.9±9.25	38.2±5.1	Adult	Chronic	NO	237.6±133.2	NA	16.0±2.1	22.1±1.4	1.0 T MRI		NA	*p*_uncorrected_ <.001	
(Brooks et al., 2011)	14(14)	21(21)	26.0±1.9	26±2.1	Adult	NA	YES	110.4±22.8	NA	15.6±0.4	21.4±0.5	1.5 T MRI	SPM5	12	NA	9.5
(Gaudio et al., 2011)	16(16)	16(16)	15.2±1.7	15.1±1.5	Adolescent	Acute	NO	5.3±3.2	14.7±1.7	14.2±1.4	20.2±1.6	1.5 T MRI	SPM2	NA	*p*_uncorrected_ <.001	10
(Joos et al., 2011)^b^	5(5)	18(18)	29.6±5.1	26.9±5.7	Adult	Recovered	NO	86.4±72	NA	19.9±1.5	21.2±2.0	3.0 T MRI	SPM8	12	*p*_uncorrected_ <.001	9
	12(12)		25.0±4.8		Adult		NO	NA	NA	16.0±1.2	21.2±2.0					
(Friederich et al., 2012)^b^	12(12)	14(14)	24.3±6.2	25.6±3.7	Adult	Acute	NO	75.6±52.8	NA	15.9±1.6	21.1±1.5	3.0 T MRI	SPM5	8	*p*_corrected_ <.05, *p*_uncorrected_ <.05	9
	13(13)		25.0±4.8		Adult	Recovered	NO	68.4±43.2	NA	19.5±1.4	21.1±1.5					
(Mainz et al., 2012)	19(19)	19(19)	15.7±1.5	15.6±1.9	Adolescent	Recovered	YES	NA	NA	15.3±1.5	21.8±2.7	3.0 T MRI	SPM5	NA	FWE corrected	9.5
(Amianto et al., 2013)	17(17)	14(14)	20.0±4.0	24.0±3.0	Adolescent	Acute	NO	13.0±8.0	NA	16.0±1.0	21±2	1.5 T MRI	FSL	7	*p*_TFCE_ <.005	9.5
(Cicerale et al., 2013)	10(10)	8(8)	22.0±4.0	24.0±2.0	Adult	NA	NA	16.0±9.0	16±9	15.9±1.0	21.1±1.7	1.5 T MRI	FSL	7	*p*_TFCE_<.005	9
(Frank, Shott, Hagman, & Mittal, 2013)	19(19)	24(24)	23.1±5.8	27.4±6.3	Adult	Acute	NO	NA	NA	16.0±1.1	21.6±1.3	3.0 T MRI	SPM8	8	*p*_FWE_ <.05	9.5
(Lazaro et al., 2013)	35(NA)	17(NA)	16.3±1.3	16.7±1.5	Adolescent	Recovered	NO	7.4±5.2	14.02±1.6	19.3±1.1	NA	1.5 T MRI	SPM8	12	*p*<.05	9
(Fonville, Giampietro, Williams, Simmons, & Tchanturia, 2014)	31(NA)	31(NA)	23.0	25.0	Adult	NA	YES	84.0±120.0	16±4.75	15.8±1.4	21.8±1.8	1.5 T MRI	FSL	7	*p*_FWE_ <.05	9
(Bar, de la Cruz, Berger, Schultz, & Wagner, 2015)	26(23)	26(23)	23.0±5.0	24.0±1.9	NA	Acute	NO	22.4±14.8	NA	17.0±1.5	21.7±1.5	1.5 T MRI	SPM8	10	*p*_uncorrected_<.001	9.5
(Bomba et al., 2015)	11(11)	8(8)	13.6±2.8	13.3±2.4	Adolescent	NA	NO	14.5±10.9	NA	12.8±0.8	19.9±1.5	1.5 T MRI	SPM5	8	*p*_FWE_ <.05	9
(D'Agata et al., 2015)	21(21)	17(17)	21.0±5.0	23.0±4.0	Adolescent	Acute	NO	NA	NA	16.1±0.9	21.5±2.3	1.5 T MRI	FSL	NA	*p*_uncorrected_<.005	9.5
(Fujisawa et al., 2015)	20(20)	14(14)	14.2±1.8	14.9±1.6	Adolescent	NA	NO	23.6±17.0	12.6±1.8	14.4±2.1	NA	3.0 T MRI	SPM8	12	L_IFG: *p*_FWE_ <.05 R_IFG: *p*_uncorrected_ <.05	9.5
(Seitz et al., 2015)	56(56)	50(50)	15.5±1.7	15.8±1.7	Adolescent	Acute	NO	11.4±8.3	14.5±1.6	15.1±1.4	21.4±3.3	3.0 T MRI	Free surfer	NA	*p*_Bonferroni_<.01	9.5
(van Opstal et al., 2015)	10(10)	11(11)	22.1±3.3	20.8±0.5	Adult	NA	NO	42.5±27.6	NA	15.6±1.0	20.3±1.5	3.0 T MRI	FSL	NA	NA	9
(Björnsdotter et al., 2017)	25(25)	25(25)	20.3±2.2	21.3±2.1	Adolescent	NA	NO	49.7±42.5	NA	16.3±0.9	21.1±2.3	3.0 T MRI	SPM8	8	*p*_corrected_ <.05	9.5
(Kohmura et al., 2017)^b^	23(23)	29(29)	28.5±6.7	28.2±7.0	Adult	NA	NO	126±74.4	18.0±3.2	13.2±1.5	21.5±3.3	3.0 T MRI	SPM8	8	*p*_FWE_ <.05	9.5
	23(23)		NA	NA	Adult	NA	NO									
(Martin Monzon et al., 2017)	26(26)	20(20)	16.5±0.3	17.3±0.4	Adolescent	NA	NO	NA	NA	16.7±0.2	22.6±0.9	3.0 T MRI	SPM12	6	*p*_FDR_ <.05	9.5
(Nickel et al., 2017)	34(34)	41(41)	23.8±4.3	23.6±3.8	Adult	Acute	NO	79.2±44.4	NA	16.1±1.4	22.3±2.4	3.0 T MRI	SPM12	8	*p*_FWE_ <.05	9.5
(Olivo et al., 2018)	22(22)	38(38)	14.9±1.6	14.7±1.3	Adolescent	Acute	NO	7.9±4.8	NA	19.3±2.0	20.7±2.3	3.0 T MRI	SPM12	8	*p*_FWE_ <.001	9.5
(Phillipou et al., 2018)	26(26)	27(27)	22.8±6.7	22.5±3.2	Adolescent	Acute	YES	6.4±7.4	16.0±3.4	16.6±1.2	22.6±3.5	3.0 T MRI	SPM12	8	*p*_FWE_<.05	9.5
(Oliva et al., 2020)	15(15)	15(15)	25.9±6.2	25.2±1.0	Adult	Recovered	NO	38.4±38.0	16.5±2.3	20.1±2.0	21.3±2.5	1.5 T MRI	SPM12	8	NA	9.5

Abbreviations: AN, anorexia nervosa patients; BMI, body mass index; c, the Threshold‐Free Cluster Enhancement; FDR, false discovery rate; FSL, FMRIB's Software Library, the University of Oxford; FWE, family wise error; FWHM, full width at half maximum; HC, healthy control; IFG, inferior frontal gyrus; L_IFG, left IFG; NA, not available; R_IFG, right IFG; SPM, statistical parametric mapping; VBM, voxel‐based morphometry.

^a^ Total score out of 10.

^b^ At least two data sets are included.

**TABLE 2:** Demographic, clinical, and imaging characteristics of the included studies of resting‐state functional activity

Study	Demographic characteristics					Clinical characteristics (AN patients)							Imaging characteristics				
	Participants (female), *n*		Mean age±*SD*, year		Age group	AN state	Comorbid	Illness duration (months)	Age at onset (years)	BMI		Imaging technique^a^	Scanner	Software	FWHM (mm)	Threshold	Quality score^b^
	AN	HC	AN	HC						AN	HC						
(Gordon et al., 2001)	8(8)	8(8)	20.1±2.6	23.7±2.6	NA	NA	NO	33.2±32.4	NA	18.0±3.5	21.7±1.8	**PET**	PET	SPM96	6	NA	8.5
(Naruo et al., 2001)	7(7)	7(7)	NA	NA	NA	NA	NO	NA	NA	12.8±2.1	20.0±1.4	**SPECT**	SPECT	SPM96	12	NA	8.5
(Takano et al., 2001)	14(14)	8(8)	21.2±6.6	28.3±3.3	NA	NA	NO	16.8±9.6	NA	14.0±2.2	19.7±0.8	**SPECT**	SPECT	SPM96	10	*p*_uncorrected_ <.05	9
(Kojima et al., 2005)^c^	12(12)	11(11)	18.6±3.5	21.8±2.1	NA	Before WG	NO	NA	NA	12.5±1.7	20.1±0.8	**SPECT**	SPECT	SPM99	12	*p*_uncorrected_ <.05	9.5
	12(12)	11(11)	18.8±3.5	21.8±2.1	NA	After WG	NO	NA	NA	15.6±0.7	20.1±0.8			NA	NA		
(Jinjue et al., 2007)	3(3)	25(25)	20.3	20.4±1.8	Adult	NA	NO	NA	NA	16.3	21.3±1.2	**SPECT**	SPECT	SPM2	NA	*p*_uncorrected_ <.05	9
(Yonezawa et al., 2008)^c^	26(26)	10(10)	22.2±3.9	20.6±1.7	NA	Acute and chronic	NO	32.0±22.4	19.7±4.3	13.5±1.3	19.7 ±1.8	**SPECT**	SPECT	NA	8	NA	9.5
	13(13)	10(10)	22.2±3.6	20.6±1.7	NA	Acute	NO	30.3±23.7	19.6±4.6	13.8±1.5	19.7 ±1.8			NA	NA	NA	
	13(13)	10(10)	22.3±4.4	20.6±1.7	NA	Chronic	NO	33.8±21.9	19.7±4.2	13.4±1.2	19.7 ±1.8			NA	NA	NA	
(Cicerale et al., 2013)	10(10)	8(8)	22.0±4.0	24.0±2.0	NA	NA	NO	16.0±9.0	20.0±4.0	15.9±1.0	21.1 ±1.7	**ALFF**	1.5T MRI	FSL	NA	*p*_TFCE_ <.005	9.0
(Pasanisi et al., 2013)^c^	7(7)	20(20)	23.4±4.5	25.6±3.9	Adult	Acute	NO	NA	NA	15.5±0.8	22.2±1.5	**PET**	PET	NA	NA	NA	8.5
	3(3)	20(20)	21.3±1.5	25.6±3.9	NA	Recovered	NO	NA	NA	18.8±1.1	22.2±1.5			NA	NA	NA	
(Yue, 2013)	8(8)	8(8)	20.2±4.8	19.7±3.5	14‐32	NA	NO	NA	NA	16.1±2.8	20.6±2.4	**ReHo**	3.0T MRI	SPM8	4	*p*_uncorrected_ <.005	8.5
(Zhang et al., 2013)	6(6)	12(12)	17.0±0.8	24.0±3.3	NA	NA	NO	23.8±10.5	NA	12.2±1.0	NA	**PET**	PET	SPM2	10	*p*_uncorrected_ <.001	9.0
(Sheng et al., 2015)^c^	23(23)	25(25)	25.8±8.3	25.5±6.6	Adult	Acute	YES	NA	NA	18.2±1.6	22.6±2.7	**ASL**	3.0T MRI	SPM5	8	*p*_FWE_<<.001	9.5
	19(19)	25(25)	30.4±8.4	25.5±6.6	Adult	Recovered	YES	NA	NA	23.0±3.0	22.6±2.7						
(Wierenga et al., 2017)	21(21)	16(16)	27.2±1.7	23.9±1.5	Adult	NA	NO	NA	NA	21.8±0.3	22.4±0.4	**ASL**	3.0T MRI	FSL	4	*p*_corrected_ <.05	9.5
(Seidel et al., 2019)^c^	74(74)	74(74)	16.0±2.9	16.2±2.9	12.1‐28.5	Acute and nonchronic	NO	17.5±22.7	NA	14.6±1.3	20.6±2.6	**fALFF**	3.0T MRI	SPM8	6	*p*_FDR_<.05	9.5
	74(74)	74(74)	16.0±2.9	16.2±2.9	NA		NO			14.5±1.3	20.6±2.6	**ReHo**	3.0T MRI	NA	NA	NA	
(Lai et al., 2020)	7(7)	14(14)	17.5±2.4	19.1±3.1	NA	Acute	NO	10.3±6.7	NA	13.7±1.4	19.6±1.8	**fALFF**	3.0T MRI	DPARSF	4	*p*_GFR_ <.05	9
(Seidel et al., 2019)	65(65)	65(65)	22.06±3.3	22.05±3.3	NA	Recovered	NO	NA	NA	20.7±1.8	21.61±2	**fALFF**	3.0T MRI	SPM8	6	*p*FWE<<.05	9.5

Abbreviations: ALFF, amplitude of low‐frequency fluctuation; AN, anorexia nervosa patients; ASL, arterial Spin Labeling; BMI, body mass index; DPARSF, data processing assistant for resting‐State fMRI software; fALFF, the fractional ALFF; FDR, false discovery rate; fMRI, functional magnetic resonance imaging; FSL, FMRIB's Software Library, the University of Oxford; FWE, family wise error; FWHM, full width at half maximum; HC, healthy control; NA, not available; ReHo, regional homogeneity; *SD*, standard deviation; SPM, statistical parametric mapping; TFCE, the threshold‐free cluster enhancement; WG, weight gain.

^a^ Include ALFF, ALFF, ReHo, PET, SPECT, and ASL.

^b^ Total score out of 10.

^c^ Represents at least two data sets are included.

### 3.2 Meta‐analysis

#### 3.2.1 Pooled meta‐analysis

##### VBM

**FIGURE 2:**
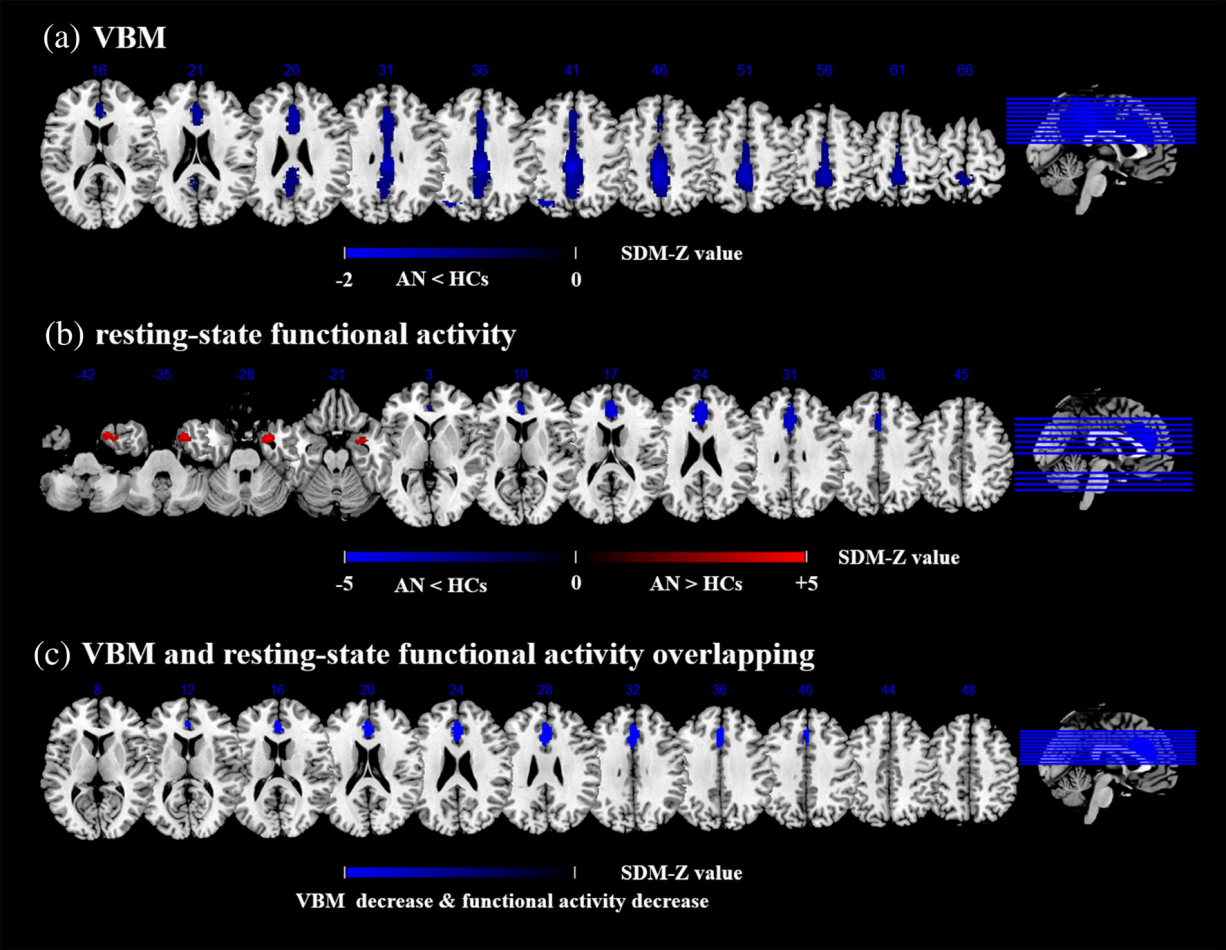
Meta‐analyses results regarding (a) GMV difference between AN and HCs, (b) Resting‐state functional activity difference between AN and HCs, (c) Conjunction of GMV differences and resting‐state functional activity differences. Areas with decreased GMV value or resting‐state functional activity value are displayed in blue, and areas with increased GMV value or resting‐state functional activity value are displayed in red. The color bar indicates the maximum and minimum SDM‐Z values. AN, anorexia nervosa; HCs, healthy controls; SDM, seed‐based d mapping; VBM, voxel‐based morphometry; GMV, gray matter volume

Structural differences in patients with AN relative to HCs are shown in Table 3 and Figure 2a. AN patients decreased GMV in the bilateral MCC (extending to the bilateral precuneus, posterior cingulate cortex [PCC], ACC, superior frontal gyrus), and left middle occipital gyrus (extending to left inferior parietal lobe). No significantly increased GMV was observed in patients with AN.

**TABLE 3:** Meta‐analyses results regarding VBM and resting‐state functional brain activity difference between AN and HCs

Local maximum				Cluster				
Region	Peak MNI coordinate (x, y, z)	SDM‐Z value	*p* value	No. of voxels	Breakdown (no. of voxels)	Egger's test (*p* value)	Jackknife sensitivity	Heterogeneity
***VBM***								
***AN* vs. *HCs (AN < HCs)*** bilateral median cingulate/paracingulate gyri, BA 23	0,−30,40	−4.727	.001	5497	Left precuneus, BA 5 (860) Right precuneus, BA 7 (503) Left anterior cingulate/paracingulate gyri, BA 32 (434)	.906	35/35	No (*I* ^2^<50%)
					Right anterior cingulate/paracingulate gyri, BA 32 (225)			
					Left median cingulate/paracingulate gyri, BA 24 (1141)			
					Right median cingulate/paracingulate gyri, BA 4 (1046)			
					Left posterior cingulate gyrus, BA 26 (272)			
					Right posterior cingulate gyrus, BA 26 (72)			
					Left superior frontal gyrus, medial (168)			
					Right superior frontal gyrus, medial (17)			
Left middle occipital gyrus, BA 7	−32,−72,38	−3.425	.027	124	Left inferior parietal gyri, BA 7 (36)	.982	35/35	No (*I* ^2^<50%)
					Left middle occipital gyrus, BA 7 (49)			
					Left superior occipital gyrus, BA 7 (15)			
					Left superior parietal gyrus, BA 7 (9)			
***Resting‐state functional activity***								
***AN* vs. *HCs (AN> HCs)*** right parahippocampal gyrus, BA 28	24,2,‐32	1.905	<.001	465	Right temporal pole, middle temporal gyrus, BA 36 (78) Right parahippocampal gyrus, BA 28 (106) Right amygdala, BA 36 (94)	.979	22/22	No (*I* ^2^<50%)
***AN* vs. *HCs (AN < HCs)*** bilateral anterior cingulate/paracingulate gyri, BA 24	0,36,20	−1.705	<.001	1317	Left anterior cingulate/paracingulate gyri, BA 32 (595) Right anterior cingulate/paracingulate gyri, BA 32 (235) Left median cingulate/paracingulate gyri, BA 24 (150) Right median cingulate/paracingulate gyri, BA 24 (99)	.084	19/22	No (*I* ^2^<50%)

Abbreviations: AN, anorexia nervosa; BA, Brodmann area; HCs, healthy controls; JK, Jackknife sensitivity analysis; MNI, Montreal Neurological Institute; SDM, the seed‐based d mapping; VBM, voxel‐based morphometry.

##### Resting‐state functional imaging

Functional differences in patients with AN relative to HCs are shown in Table 3 and Figure 2b. AN patients displayed decreased resting‐state functional activity in the bilateral anterior cingulate cortex (ACC) (extending to the bilateral superior frontal gyrus) and bilateral median cingulate cortex (MCC), and increased activity in the right parahippocampal gyrus (extending to the right temporal pole, middle temporal gyrus, and amygdala).

#### 3.2.2 Subgroup meta‐analysis

##### VBM

The adult AN subgroup (included 20 data sets) demonstrated decreased GMV in the bilateral ACC (extending to the bilateral MCC, superior frontal gyrus, and superior frontal gyrus). The female AN patient subgroup (included 29 data sets) showed the same clusters of GMV variation as the adult AN patient subgroup. The acute AN patient subgroup (included 11 data sets) displayed decreased GMV in the bilateral MCC. The subgroup of AN with no psychiatric comorbidities (included 30 data sets) showed decreased GMV in the bilateral MCC (extending to the bilateral ACC, PCC, and the supplementary motor area). These results are in agreement with the pooled meta‐analysis, indicating that the main effects are stable and reliable (Tables S1–S4). However, there were too few resting‐state functional imaging studies to perform the following subgroup: functional brain activity of adult and untreated AN patients.

##### Resting‐state functional imaging

The subgroup of AN with no psychiatric comorbidities (included 19 data sets) demonstrated decreased resting‐state functional activity in the bilateral ACC (extending to the bilateral MCC and left superior frontal gyrus), and increased activity in the right amygdala (extending to the right temporal pole and parahippocampal gyrus) (Table S6).

#### 3.2.3 Sensitivity analysis

As seen in Table 3, the whole‐brain jackknife sensitivity analysis revealed that, the most replicable findings in patients with AN were the decrease in GMV in the bilateral MCC (extending to the bilateral ACC and PCC) and left inferior parietal lobe (which were replicable in all 34 data sets). In addition, the resting‐state functional activity decrease in the bilateral anterior cingulate cortex and bilateral median cingulate cortex, and increase in the right parahippocampal gyrus (preserved in at least 85% of the combinations).

#### 3.2.4 Analyses of heterogeneity and publication bias

##### VBM

The low *I* ^2^ statistic indicates very small heterogeneity, the funnel plot does not show asymmetries (Figure S1A,B) and the Egger's test did not show any publication bias for these areas (Egger's *p* = .906, .982).

##### Resting‐state functional imaging

The *I* ^2^ statistic indicates a certain extent heterogeneity. The funnel plot is listed in Figure S1C,D, and the Egger's tests show no publication bias in these areas (Egger's *p* = .979, .084).

#### 3.2.5 Meta‐regression analyses

Illness duration, age, and BMI were not associated with AN‐related GMV or resting‐state functional activity changes. We were unable to assess the relationship to AN symptom severity because this was reported using a variety of incompatible measures.

#### 3.2.6 Conjunction analyses

A conjunction analysis further found that the ACC and MCC were altered in AN in both VBM and resting‐state functional studies. Specifically, AN patients showed concurrent decreased GMV and hypoactivity in these regions (Figure 2c).

## 4 DISCUSSION

To the best of our knowledge, this is the first whole‐brain voxel‐based meta‐analysis of all available VBM and resting‐state functional imaging studies on AN. The main findings were as follows: (a) AN patients showed reduced GMV of the bilateral MCC (extending to the ACC and PCC), and left middle occipital gyrus (extending to left inferior parietal lobe). (b) AN patients showed decreased resting‐state functional activity in the bilateral ACC and MCC, and increased activity in the right parahippocampal gyrus. (c) structural and functional reductions overlapped in the bilateral ACC and MCC in the patients with AN. Additionally, meta‐regression analyses demonstrated that the main resting‐state functional activity and GMV changes in AN remained unaffected by the potential confounding variables of illness duration and BMI, which further confirm the robustness of the findings. These findings provide novel insights into the pathophysiology of AN.

This study, found reduced GMV and spontaneous neural activity in the cingulate cortex (particularly in the ACC and MCC) in AN, suggesting both structural and functional impairments in these regions in AN. The findings of reduced GMV in the ACC and MCC are partly consistent with a previous meta‐analysis that showed decreased GMV in the bilateral MCC and PCC (Zhang et al., 2018). The minor difference may be due to the larger number of studies and more accurate methodology used in this meta‐analysis. Additionally, a previous meta‐analysis of task‐based fMRI studies reported abnormal activity in the ACC in AN patients when receiving food stimuli (Zhu et al., 2012), which further supports our findings of abnormal regional function in the brain ACC of AN patients. The ACC is a part of a circuit involved in attention that serves to regulate both cognitive and emotional processing (Bush, Luu, & Posner, 2000), and appears to play a crucial role in initiation, motivation, and goal‐directed behaviors (Devinsky, Morrell, & Vogt, 1995). A task‐based fMRI study showed that increased rCBF in the ACC was associated with anxiety and physiological arousal in AN, and the AN group showed higher level of anxiety along with increased blood flow in the ACC in response to contrasting stimuli (Ellison et al., 1998). However, more studies are needed to confirm the neural activity variations between resting and task‐dependent states of AN. Also, the ACC has been suggested to be specifically involved in body image distortion (Friederich et al., 2010; Gaudio et al., 2015), abnormal reward processing (Kaye, Fudge, & Paulus, 2009; Keating, 2011), impaired cognitive‐behavioral flexibility (Zastrow et al., 2009), excessive cognitive control of appetite (Kim, Ku, Lee, Lee, & Jung, 2012), and perfectionism (Kaye et al., 2009) in AN patients. Given these considerations, the disturbances in this area might seriously alter the perception/conception and emotional regulation in the patients with AN. Additionally, a longitudinal study found reduced ACC volume during active AN, which was normalized with weight restoration, and smaller changes in right dorsal ACC volume prospectively predicted relapse after treatment (McCormick et al., 2008). Therefore, our findings provide a potential brain‐specific marker for the treatment and outcomes of patients with AN.

The MCC is involved in negative cognition and emotion regulation (Jiang, He, Guo, & Gao, 2019; Riemann et al., 2010), especially the emotionally salient stimuli processing (Maddock, 1999; Small, Zatorre, Dagher, Evans, & Jones‐Gotman, 2001). In the present study, the results of the two meta‐analyses overlapped in the MCC (showing both GMV reductions and functional hypoactivity in AN as compared with HCs), suggesting that structural lesions in the MCC might be linked to functional impairment. The aberrant structure and neural activity of the MCC has been hypothesized to be linked to excessive perfectionism (Fassino et al., 2006), cognitive rigidity, and excessive attention to detail (Friederich & Herzog, 2011). This anorectic personality type relates to exaggerated cognitive control, impaired cognitive set‐shifting (i.e., concrete and rigid behaviors to changing rules), and impaired behavioral response shifting (i.e., stereotyped or perseverative behaviors) (Fairburn, Shafran, & Cooper, 1999; Roberts, Tchanturia, Stahl, Southgate, & Treasure, 2007). This might explain the body image distortion of AN patients that leads to excessive concern about weight (even when the patients have seriously low body weight) and body shape (Fairburn & Harrison, 2003). Additionally, the MCC is also related to energy‐sensing. It has been found that different taste activities with different energy contents during starvation differ from those during satiety (van Rijn, de Graaf, & Smeets, 2015). Therefore, the MCC might play an important role in mental representation, body image, and energy‐sensing (Gaudio et al., 2011) in AN. Taken together, the structural and functional disruptions of the ACC and MCC may be a crucial neurobiological feature of AN.

Specific to VBM, we observed decreased GMV in the left middle occipital gyrus (extending to left inferior parietal lobe), and precuneus/PCC in AN, which were partially consistent with the two previous meta‐analyses of VBM studies (Titova et al., 2013; Zhang et al., 2018). However, this was the largest VBM meta‐analysis on AN (including 660 patients with AN, and 740 controls), with stricter correction. The occipital cortex plays an important role in processing visual information that is subsequently transferred to the temporal cortex for further integration (Amianto et al., 2013; Madsen, Bohon, & Feusner, 2013). Specifically, recent investigations have linked body image distortions in AN to the posterior part of the fusiform gyrus (Suchan et al., 2010; Suchan et al., 2013). These findings showed the possibility that deficit in the middle occipital gyrus could be associated with the body image distortion experienced by individuals with AN. Moreover, our meta‐analysis found decreased GMV in the left inferior parietal lobe (which extended from the left middle occipital gyrus) and precuneus/PCC in AN. The inferior parietal lobe and precuneus/PCC are core hubs of the default mode network (DMN), which plays an important role in cognitive performance to balance external stimulus processing and self‐directed processing (Gerlach, Spreng, Madore, & Schacter, 2014; Price & Drevets, 2010). Several rs‐fMRI studies found abnormal functional connectivity in the DMN in acute and recovered patients with AN (Boehm et al., 2014; Cowdrey, Filippini, Park, Smith, & McCabe, 2014; McFadden, Tregellas, Shott, & Frank, 2014), and the DMN dysfunction might be related to the core symptoms of AN, including ruminative preoccupation with eating, body weight and shape, excessive planning and impaired cognitive flexibility (Cowdrey et al., 2014; Kaye et al., 2009). Additionally, the parietal cortex is related to abnormal food intake rewards (e.g., long‐term restricted food intake may lead to abnormal reward response and unpleasant sense of normal eating) and satiation in AN (Decker, Figner, & Steinglass, 2015). Therefore, the findings of gray matter atrophy in the left middle occipital gyrus (extending to left inferior parietal lobe) and precuneus/PCC could be associated with the core symptoms in AN, which suggests that the DMN dysfunction might be a vulnerability marker for the development of AN.

Additionally, the present meta‐analysis found increased resting‐state functional activity in the right parahippocampal gyrus in AN. Several structural MRI studies found reduced parahippocampal volumes in AN (Brooks et al., 2011; Connan et al., 2006). The parahippocampal gyrus is a limbic system structure, located in the medial temporal lobe. It is involved in the formation and regulation of learning, memory, and emotional control of food intake (Aminoff, Kveraga, & Bar, 2013; Rosenbaum, Sy, Pavlovich, Leibel, & Hirsch, 2008; Squire et al., 2010) such as the memory of specific attributes of recently eaten food (Higgs, 2008). Task‐based fMRI studies found abnormal activity in the parahippocampal gyrus in AN patients during viewing their own bodies (Vocks et al., 2010), perception of distorted body images (Miyake et al., 2010), and cognitive flexibility on the Wisconsin Card Sorting Test (WCST) (Sato et al., 2013). Taken together, these findings of spontaneous functional activity abnormalities in the parahippocampal gyrus may contribute to the rigid and strategic cognitive styles associated with AN. However, the findings need to be verified by further homogeneity studies with large samples.

## 5 LIMITATIONS

First, this study was based on summarized data (e.g., meta‐analyses of peak and effect sizes from published studies) rather than raw data (Radua et al., 2012), and few small or moderate effect size results may have been neglected. Second, the studies included in this meta‐analysis used different statistical thresholds and multiple comparison corrections. Third, some studies included in the present analyses did not rigorously adhere to the guidelines for brain imaging studies in eating disorders (Frank, Favaro, Marsh, Ehrlich, & Lawson, 2018). Therefore, the clinical characteristics such as age (adolescents or adults), BMI, duration of illness, AN status (acute, chronic, and recovery), age of onset, eating disorder‐related cognitions, depression severity, and inclusion of both binge/purge and restricting types of AN (Kaufmann et al., 2020; Seitz, Herpertz‐Dahlmann, & Konrad, 2016), may act as potential confounding factors. Fourth, many subjects were taking psychotropic medicines such as antidepressants, so whether the alteration of cerebral functional activity and GMV in AN participants were caused by medicines or by AN remains unknown. Fifth, there may have heterogeneity in the results of functional analyses, because they were based on several different measures (ReHo, ALFF, ASL, PET, SPECT). Therefore, we have carried out the subgroup analysis of AN patients in SPECT studies (included 8 data sets) (Table S7, Figure S8) and the results (bilateral anterior cingulate/paracingulate gyri) validated the main results. Finally, the results may be generalized to females but not males because the majority of studies included female participants.

## 6 CONCLUSION

By summarizing functional and structural studies to date, this meta‐analysis demonstrated a significant reduction in the functional activity and gray matter in the cingulate cortex in patients with AN, particularly in the ACC and MCC, which imply that structural changes may underlie functional alterations. These results expand the current understanding of functional and structural brain abnormalities in AN patients, which would provide additional potential targets for therapeutic intervention.

## CONFLICT OF INTERESTS

The authors declare that they have no conflict of interests.

## AUTHOR CONTRIBUTIONS

Ting Su: conception and design, acquisition of data, analysis and interpretation of data; drafted the article. Jiaying Gong: conception and design, acquisition of data. Guixian Tang: analysis and interpretation of data; drafted the article. Shaojuan Qiu: revised the manuscript critically for important intellectual content. Pan Chen: revised the manuscript critically for important intellectual content. Guanmao Chen: acquisition of data. Junjing Wang: acquisition of data. Li Huang: revised the manuscript critically for important intellectual content. Ying Wang: conception and design, acquisition of data, analysis and interpretation of data; revised the manuscript critically for important intellectual content. All authors gave final approval for the version to be published.

## Supporting information

**Appendix S1** Supplementary Information.

## Data Availability

The data that support the findings of this study are available from the corresponding author upon reasonable request.
