# Supplementary material for: Structural and functional brain alterations in anorexia nervosa:A multimodal meta‐analysis of neuroimaging studies
Source: Hum Brain Mapp. 2021 Jul 23;42(15):5154–69. doi: 10.1002/hbm.25602 (PMC8449099; doi:10.1002/hbm.25602)
Supplement: Supplementary file 1 — Appendix S1 Supplementary Information. [file HBM-42-5154-s001.docx]

**CONTENTS**

**Table S1.** VBM results of AN subgroup (Adult)

**Table S2.** VBM results of AN subgroup (Female)

**Table S3.** VBM results of AN subgroup (Acute AN)

**Table S4.** VBM results of AN subgroup (AN with no psychiatric comorbidities)

**Table S5.** VBM results of AN subgroup (excluding studies with small samples [n<10])

**Table S6.** Resting-state functional activity results in AN subgroup (AN with no psychiatric comorbidities)

**Table S7.** Resting-state functional activity results in AN subgroup (SPECT)

**Table S8.** Resting-state functional activity results in AN subgroup (excluding studies with small samples [n<10])

**Table S9.**Quality assessment checklist (score 0/0.5/1 per item; total score out of 10)

**Figure S1.** Funnel plot for the peak of the cluster of statistically significantly smaller gray matter volume, hyperperfusion, and hypoperfusion.

**Figure S2.** VBM results of AN subgroup (Adult)

**Figure S3.** VBM results of AN subgroup (Female)

**Figure S4.** VBM results of AN subgroup (Acute AN)

**Figure S5.** VBM results of AN subgroup (AN with no psychiatric comorbidities)

**Figure S6.** VBM results of AN subgroup (excluding studies with small samples [n<10])

**Figure S7.** Resting-state functional activity results in AN subgroup (AN with no psychiatric comorbidities)

**Figure S8.** Resting-state functional activity results in AN subgroup (SPECT)

**Figure S9.** Resting-state functional activity results in AN subgroup (excluding studies with small samples [n<10])

**Table S1** VBM results of AN subgroup (Adult)

| **Local Maximum** |  |  |  | **Cluster** |  |  |
| --- | --- | --- | --- | --- | --- | --- |
| **Region** | **Peak MNI coordinate**  **(x, y, z)** | **SDM-Z value** | ***p* value** | **No. of voxels** | **Breakdown (No. of voxels)** | **Egger’s test (*p* value)** |
| ***AN < HCs*** |  |  |  |  |  |  |
| Bilateral median cingulate / paracingulate gyri, BA 24 | -2,28,32 | -3.879 | 0.001 | 1426 | Left anterior cingulate / paracingulate gyri, BA 32 (521)  Right anterior cingulate / paracingulate gyri, BA 32 (273)  Left median cingulate / paracingulate gyri, BA 24 (876)  Right median cingulate / paracingulate gyri, BA 24 (911)  Left superior frontal gyrus, medial, BA 8 (338)  Right superior frontal gyrus, medial, BA 10 (107)  Right precuneus, BA 5 (55)  Left posterior cingulate gyrus, BA 23 (7) | 0.541 |

Abbreviations: HCs=healthy controls; AN=anorexia nervosa; VBM=voxel-based morphometry; MNI=Montreal Neurological Institute; SDM= seed-based d mapping; BA=Brodmann area.

**Table S2** VBM results of AN subgroup (Female)

| **Local Maximum** |  |  |  | **Cluster** |  |  |
| --- | --- | --- | --- | --- | --- | --- |
| **Region** | **Peak MNI coordinate**  **(x, y, z)** | **SDM-Z value** | ***p* value** | **No. of voxels** | **Breakdown (No. of voxels)** | **Egger’s test (*p* value)** |
| ***AN < HCs*** |  |  |  |  |  |  |
| Bilateral anterior cingulate / paracingulate gyri | 0,30,28 | -3.857 | 0.001 | 2486 | Left anterior cingulate / paracingulate gyri, BA 10 (424)  Right anterior cingulate / paracingulate gyri, BA 32 (199)  Left median cingulate / paracingulate gyri, BA 24 (286)  Right median cingulate / paracingulate gyri, BA 24 (325)  Left superior frontal gyrus, medial, BA 8 (138)  Right superior frontal gyrus, medial, BA 8 (15) | 0.682 |

Abbreviations: HCs=healthy controls; AN=anorexia nervosa; VBM=voxel-based morphometry; MNI=Montreal Neurological Institute; SDM= seed-based d mapping; BA=Brodmann area.

**Table S3** VBM results of AN subgroup (Acute AN)

| **Local Maximum** |  |  |  | **Cluster** |  |  |
| --- | --- | --- | --- | --- | --- | --- |
| **Region** | **Peak MNI coordinate**  **(x, y, z)** | **SDM-Z value** | ***p* value** | **No. of voxels** | **Breakdown (No. of voxels)** | **Egger’s test (*p* value)** |
| ***AN < HCs*** |  |  |  |  |  |  |
| Bilateral median cingulate / paracingulate gyri, BA 23 | -2,-32,40 | -4.195 | 0.002 | 522 | Left median cingulate / paracingulate gyri BA 23 (280)  Right median cingulate / paracingulate gyri, BA 23 (173) | 0.718 |

Abbreviations: HCs=healthy controls; AN=anorexia nervosa; VBM=voxel-based morphometry; MNI=Montreal Neurological Institute; SDM= seed-based d mapping; BA=Brodmann area.

**Table S4** VBM results of AN subgroup (AN with no psychiatric comorbidities)

| **Local Maximum** |  |  |  | **Cluster** |  |  |
| --- | --- | --- | --- | --- | --- | --- |
| **Region** | **Peak MNI coordinate**  **(x, y, z)** | **SDM-Z value** | ***p* value** | **No. of voxels** | **Breakdown (No. of voxels)** | **Egger’s test (*p* value)** |
| ***AN < HCs*** |  |  |  |  |  |  |
| Left median cingulate / paracingulate gyri, BA 23 | 0,-30,40 | -4.037 | 0.001 | 3480 | Left median cingulate / paracingulate gyri, BA 23 (972)  Right median cingulate / paracingulate gyri, BA 23 (904)  Left anterior cingulate / paracingulate gyri, BA 24 (461)  Right anterior cingulate / paracingulate gyri, BA 24 (207)  Left posterior cingulate gyrus, BA 23 (126)  Right posterior cingulate gyrus, BA 26 (28)  Left superior frontal gyrus, medial, BA 32 (120) | 0.981 |

Abbreviations: HCs=healthy controls; AN=anorexia nervosa; VBM=voxel-based morphometry; MNI=Montreal Neurological Institute; SDM= seed-based d mapping; BA=Brodmann area.

**Table S5** VBM results of AN subgroup (excluding studies with small samples [n<10])*

| **Local Maximum** |  |  |  | **Cluster** |  |
| --- | --- | --- | --- | --- | --- |
| **Region** | **Peak MNI coordinate**  **(x, y, z)** | **SDM-Z value** | ***p* value** | **No. of voxels** | **Breakdown (No. of voxels)** |
| ***AN < HCs*** |  |  |  |  |  |
| Left median cingulate / paracingulate gyri | -2,-38,46 | -4.069 | <0.001 | 4902 | Left median cingulate / paracingulate gyri, BA 23 (1086)  Right median cingulate / paracingulate gyri, BA 23 (963)  Left anterior cingulate / paracingulate gyri, BA 24 (409)  Right anterior cingulate / paracingulate gyri, BA 24 (156)  Left posterior cingulate gyrus, BA 23 (170)  Right posterior cingulate gyrus, BA 26 (43)  Left superior frontal gyrus, medial, BA 32 (100)  Right superior frontal gyrus, medial, BA 32 (6) |
| Right superior temporal gyrus, BA 22 | 52,-28,-2 | -2.876 | <0.001 | 610 | Right middle temporal gyrus, BA 21 (316)  Right superior temporal gyrus, BA 21 (242) |
| Right rolandic operculum, BA 48 | 38,-18,16 | -2.846 | <0.001 | 403 | Right rolandic operculum, BA 48 (151)  Right insula, BA 48 (139)  Right heschl gyrus, BA 48 (81v  Right superior temporal gyrus, BA 48 (10) |
| Left middle occipital gyrus, BA 7 | -28,-72,40 | -2.770 | <0.001 | 343 | Left middle occipital gyrus, BA 19 (132)  Left superior occipital gyrus, BA 19 (76)  Left angular gyrus, BA 7 (26)  Left inferior parietal, BA 7 (64) |

Abbreviations: HCs=healthy controls; AN=anorexia nervosa; VBM=voxel-based morphometry; MNI=Montreal Neurological Institute; SDM= seed-based d mapping; BA=Brodmann area.

*（threshold it using a p-value = 0.005, z = 1 and 50 voxels extent)

**Table S6** Resting-state functional activity results in AN subgroup (AN with no psychiatric comorbidities)

| **Local Maximum** |  |  |  | **Cluster** |  |  |
| --- | --- | --- | --- | --- | --- | --- |
| **Region** | **Peak MNI coordinate**  **(x, y, z)** | **SDM-Z value** | ***p* value** | **No. of voxels** | **Breakdown (No. of voxels)** | **Egger’s test (*p* value)** |
| ***AN*** *>* ***HCs*** | | | | | | |
| Right amygdala, BA 36 | 28,2,-28 | 1.964 | 0.005 | 166 | Right amygdala, BA 36 (76)  Right temporal pole, superior temporal gyrus, BA 38 (24)  Right parahippocampal gyrus, BA 28 (37) | 0.591 |
| ***AN < HCs*** | | | | | | |
| Left anterior cingulate / paracingulate gyri, BA 32 | 0,40,18 | -1.895 | <0.001 | 1401 | Left anterior cingulate / paracingulate gyri, BA 32 (598)  Right anterior cingulate / paracingulate gyri, BA 32 (254)  Left superior frontal gyrus, medial, BA 32 (253)  Left median cingulate / paracingulate gyri, BA 24 (154)  Right median cingulate / paracingulate gyri, BA 24 (121) | 0.176 |

Abbreviations: HCs=healthy controls; AN=anorexia nervosa; MNI=Montreal Neurological Institute; SDM= seed-based d mapping; BA=Brodmann area.

**Table S7** Resting-state functional activity results in AN subgroup (SPECT)

| **Local Maximum** |  |  |  | **Cluster** |  |  |
| --- | --- | --- | --- | --- | --- | --- |
| **Region** | **Peak MNI coordinate**  **(x, y, z)** | **SDM-Z value** | ***p* value** | **No. of voxels** | **Breakdown (No. of voxels)** | **Egger’s test (*p* value)** |
| ***AN < HCs*** |  |  |  |  |  |  |
| Left anterior cingulate / paracingulate gyri | 0,40,12 | -3.435 | <0.001 | 6141 | Left anterior cingulate / paracingulate gyri, BA 32 (1084)  Right anterior cingulate / paracingulate gyri, BA 32 (783)  Left median cingulate / paracingulate gyri, BA 24 (1170)  Right median cingulate / paracingulate gyri, BA 23 (1045)  Left posterior cingulate / paracingulate gyri, BA 23 (92)  Right posterior cingulate / paracingulate gyri, BA 23 (15)  Left superior frontal gyrus, medial, BA 32 (912)  Right superior frontal gyrus, medial, BA 32 (275)  Left supplementary motor area, BA 32 (111)  Right supplementary motor area, BA 4 (46) | 0.786 |
| Cerebellum, vermic lobule III | 0,-34,-10 | -4.598 | <0.001 | 894 | Cerebellum, vermic lobule III, BA 30 (120)  Cerebellum, vermic lobule IV / V (37)  Left cerebellum, hemispheric lobule III, BA 30 (23)  Right cerebellum, hemispheric lobule III, BA 27 (12)  Left cerebellum, hemispheric lobule IV / V, BA 30 (67)  Right lingual gyrus, BA 27 (40) | 0.723 |
| Right striatum | 4,2,-18 | -3.183 | <0.001 | 177 | Left striatum (23)  Right striatum (14)  Left olfactory cortex, BA 25 (6) | 0.801 |

Abbreviations: HCs=healthy controls; AN=anorexia nervosa; MNI=Montreal Neurological Institute; SDM= seed-based d mapping; BA=Brodmann area.

**Table S8** Resting-state functional activity results in AN subgroup (excluding studies with small samples [n<10])

| **Local Maximum** |  |  |  | **Cluster** |  |
| --- | --- | --- | --- | --- | --- |
| **Region** | **Peak MNI coordinate**  **(x, y, z)** | **SDM-Z value** | ***p* value** | **No. of voxels** | **Breakdown (No. of voxels)** |
| ***AN > HCs*** |  |  |  |  |  |
| Right inferior temporal gyrus, BA 20 | 32,2,-42 | 1.982 | 0.001 | 251 | Right fusiform gyrus, BA 36 (60)  Right temporal pole, middle temporal gyrus, BA 36 (35)  Right inferior temporal gyrus, BA 36 (21)  Right parahippocampal gyrus, BA 36 (8) |
| Right superior temporal gyrus, BA 22 | 56,-6,-10 | 1.879 | 0.002 | 167 | Right superior temporal gyrus, BA 22 (54)  Right temporal pole, superior temporal gyrus, BA 38 (27)  Right temporal pole, middle temporal gyrus, BA 21 (22)  Right insula, BA 48 (13) |
| Left thalamus | 4,-24,10 | 2.315 | <0.001 | 149 | Left thalamus (65)  Right thalamus (47) |
| Right cerebellum, crus I, BA 18 | 6,-90,-18 | 2.242 | <0.001 | 145 | Right cerebellum, crus I, BA 18 (33)  Right cerebellum, hemispheric lobule VI, BA 18 (7) |
| ***AN < HCs*** |  |  |  |  |  |
| Left anterior cingulate / paracingulate gyri | 0,28,26 | -1.742614 | 0.001 | 443 | Left anterior cingulate / paracingulate gyri, BA 24 (257)  Right anterior cingulate / paracingulate gyri, BA 24 (65)  Left median cingulate / paracingulate gyri, BA 24 (52)  Right median cingulate / paracingulate gyri, BA 24 (30)  Left superior frontal gyrus, medial, BA 32 (35) |
| Left median cingulate / paracingulate gyri, BA 23 | 0,-26,38 | -1.80985 | 0.001 | 426 | Left median cingulate / paracingulate gyri, BA 23 (264)  Right median cingulate / paracingulate gyri, BA 23 (127) |
| Right striatum | 0,4,-20 | -2.18978 | <0.001 | 85 | Left striatum (6) |
| Left precentral gyrus, BA 6 | -34,-6,54 | -1.703 | <0.001 | 78 | Left precentral gyrus, BA 6 (29)  Left superior frontal gyrus, dorsolateral, BA 6 (22)  Left middle frontal gyrus, BA 6 (27) |

Abbreviations: HCs=healthy controls; AN=anorexia nervosa; MNI=Montreal Neurological Institute; SDM= seed-based d mapping; BA=Brodmann area.

*（threshold it using a p-value = 0.005, z = 1 and 50 voxels extent)

**Table S9** Quality assessment checklist (score 0/0.5/1 per item; total score out of 10)*.

| Category 1: Participants |
| --- |
| 1. Patients were evaluated prospectively, specific diagnostic criteria were applied, and demographic data were reported. |
| 2. Healthy comparison participants were evaluated prospectively, psychiatric and medical illnesses were excluded. |
| 3. Important variables (e.g., age, sex, illness duration, onset, medication status, comorbidity, severity of illness) were checked either by stratification or statistically. |
| 4. Sample size per group > 10. |
| Category 2: Methods for image acquisition and analysis |
| 5. Whole brain analysis was automated with no a priori regional selection. |
| 6. Coordinates reported in a standard space. |
| 7. The imaging technique used was clearly described so that it could be reproduced. |
| 8. Measurements were clearly described so that they could be reproduced. |
| Category 3: Results and conclusions |
| 9. Statistical parameters for significant and important nonsignificant differences were provided. |
| 10. Conclusions were consistent with the results obtained and the limitations were discussed. |
| *When criteria were partially met, 0.5 points were awarded. |


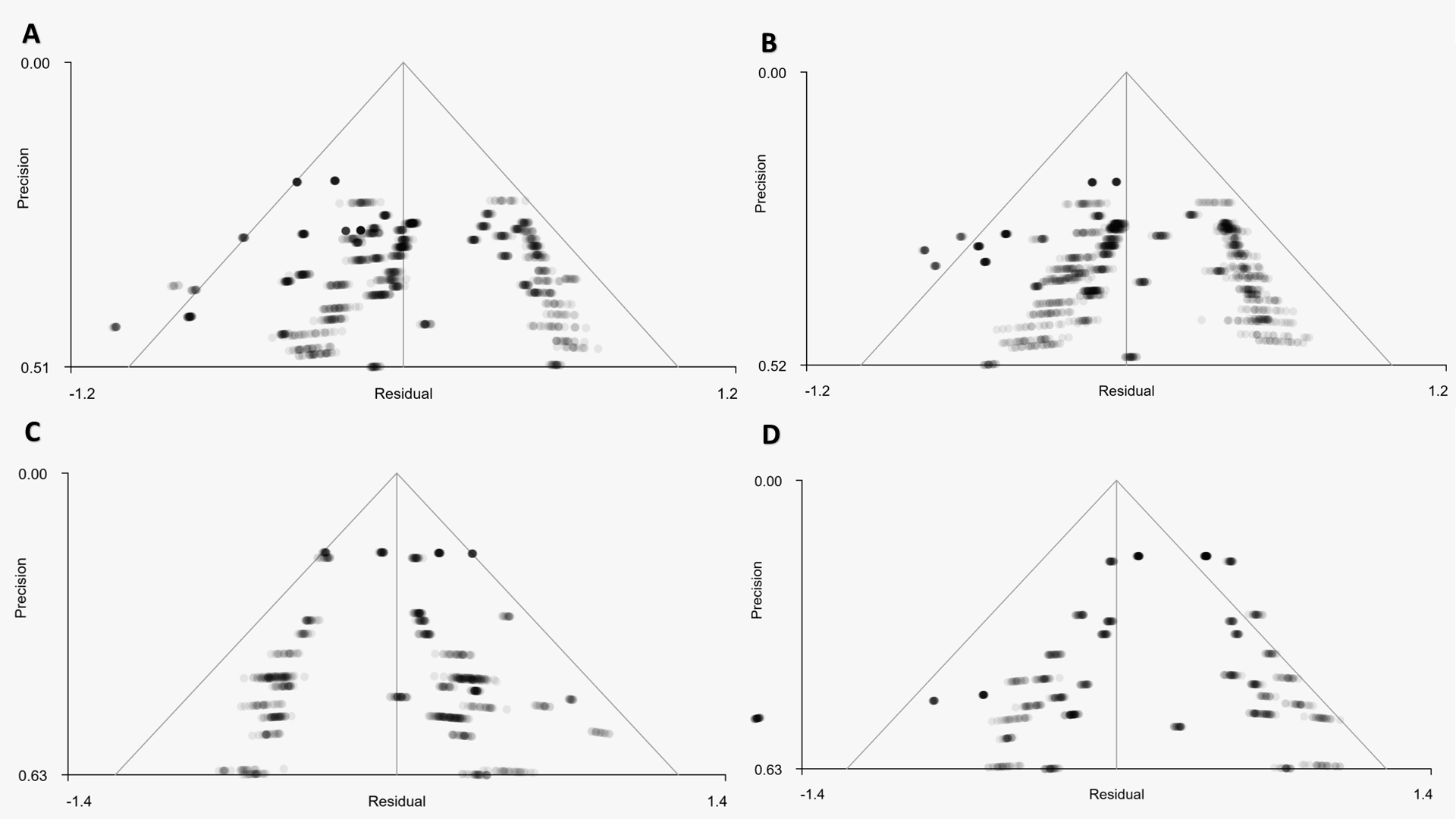


**Figure S1** Funnel plot for the peak of the cluster of statistically significantly smaller gray matter volume, hyperperfusion, and hypoperfusion.

**A** for the peak of the cluster of the bilateral median cingulate/paracingulate gyri; **B** for the peak of the cluster of the left middle occipital gyrus.

**C** for the peak of the cluster of the right parahippocampal gyrus; **D** for the peak of the cluster of the bilateral anterior cingulate/paracingulate gyri;


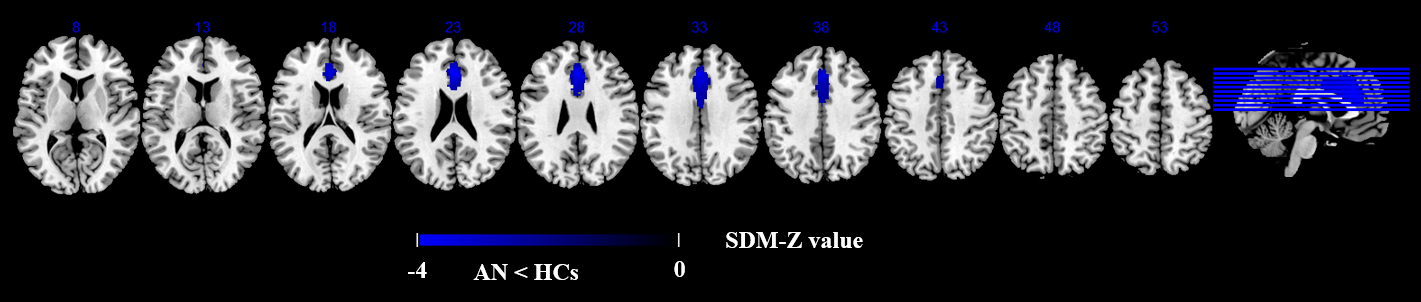
**Figure S2.** VBM results of AN subgroup (Adult). Areas with decreased GMV value. The color bar indicates the maximum and minimum SDM-Z values. Abbreviations: AN=anorexia nervosa; HCs=healthy controls; SDM=seed-based d mapping; VBM=Voxel-Based Morphometry; GMV=gray matter volume;


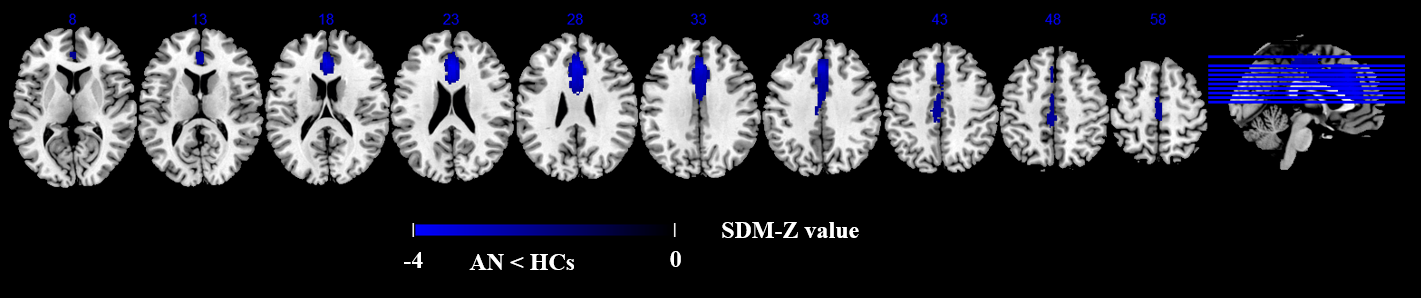
**Figure S3.** VBM results of AN subgroup (Female). Areas with decreased GMV value. The color bar indicates the maximum and minimum SDM-Z values. Abbreviations: AN=anorexia nervosa; HCs=healthy controls; SDM=seed-based d mapping; VBM=Voxel-Based Morphometry; GMV=gray matter volume;


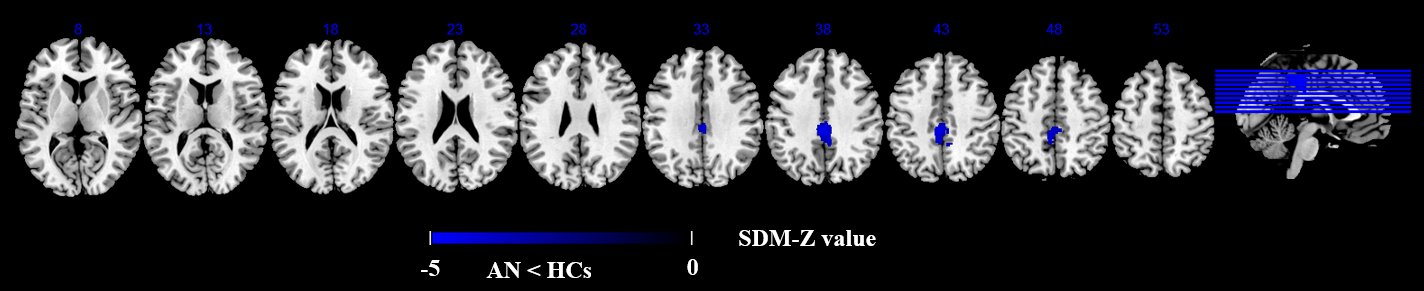
 **Figure S4.** VBM results of AN subgroup (Acute AN). Areas with decreased GMV value. The color bar indicates the maximum and minimum SDM-Z values. Abbreviations: AN=anorexia nervosa; HCs=healthy controls; SDM=seed-based d mapping; VBM=Voxel-Based Morphometry; GMV=gray matter volume;


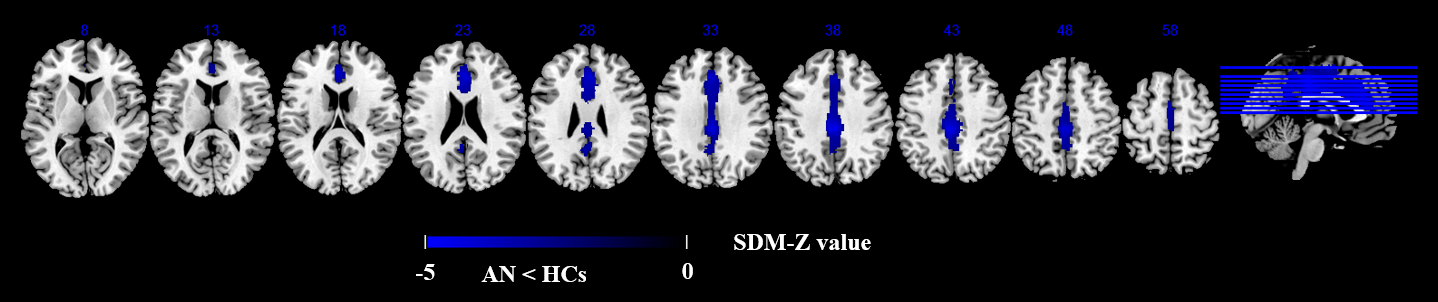
**Figure S5.** VBM results of AN subgroup (AN with no psychiatric comorbidities). Areas with decreased GMV value. The color bar indicates the maximum and minimum SDM-Z values. Abbreviations: AN=anorexia nervosa; HCs=healthy controls; SDM=seed-based d mapping; VBM=Voxel-Based Morphometry; GMV=gray matter volume;


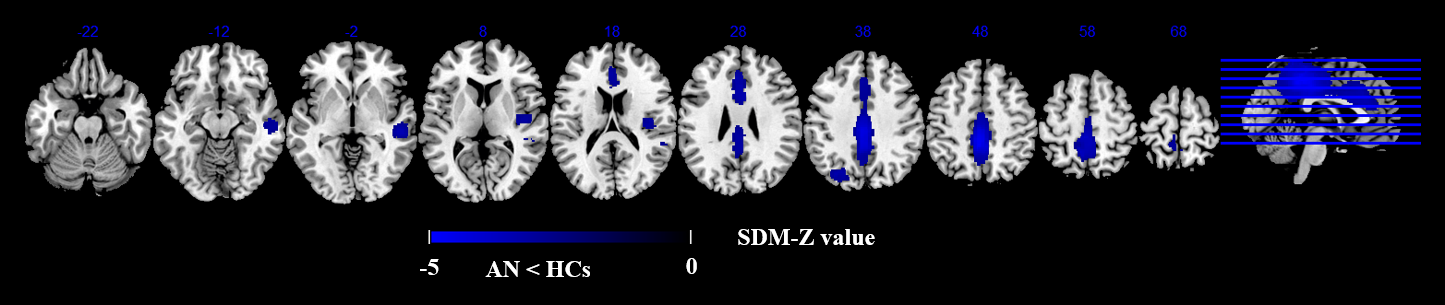
 **Figure S6.** VBM results of AN subgroup (excluding studies with small samples [n<10]). Areas with decreased GMV value are displayed in blue. The color bar indicates the maximum and minimum SDM-Z values. Abbreviations: AN=anorexia nervosa; HCs=healthy controls; SDM=seed-based d mapping; VBM=Voxel-Based Morphometry; GMV=gray matter volume;


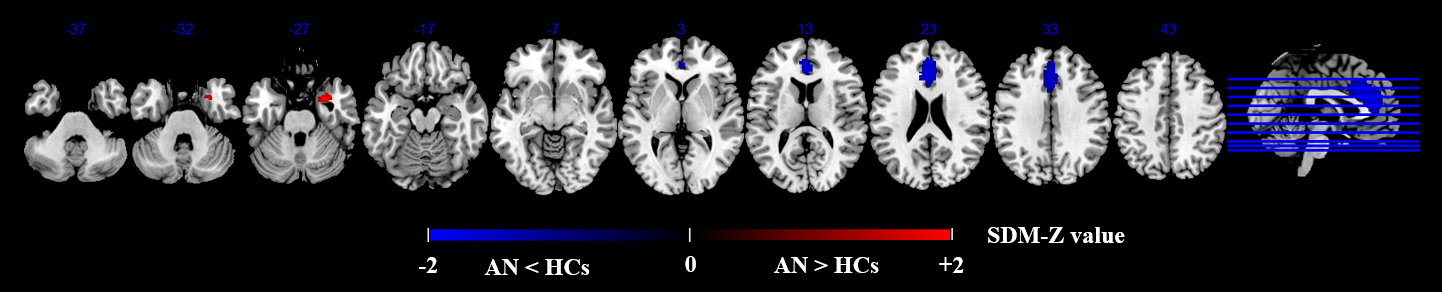
 **Figure S7.** Resting-state functional activity results in AN subgroup (AN with no psychiatric comorbidities). Areas with decreased resting-state functional activity value are displayed in blue, and areas with increased resting-state functional activity value are displayed in red. The color bar indicates the maximum and minimum SDM-Z values. Abbreviations: AN=anorexia nervosa; HCs=healthy controls; SDM=seed-based d mapping;


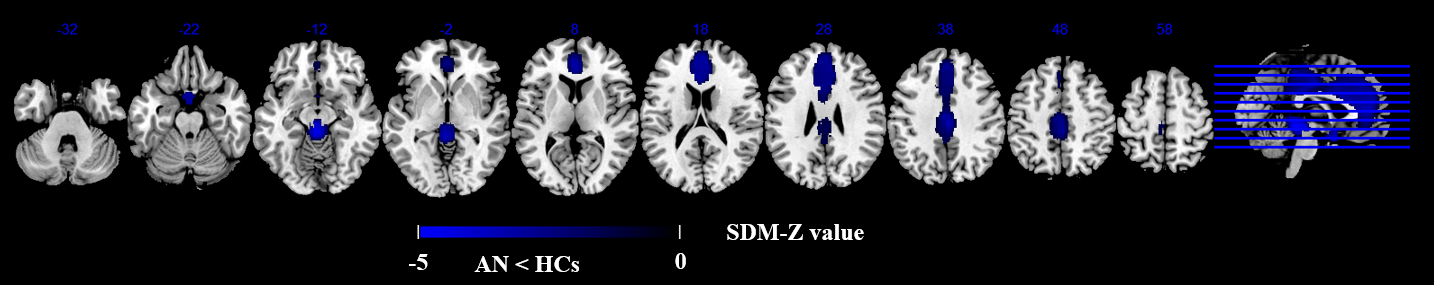


**Figure S8.** Resting-state functional activity results in AN subgroup (SPECT). Areas with decreased resting-state functional activity value are displayed in blue. The color bar indicates the maximum and minimum SDM-Z values. Abbreviations: AN=anorexia nervosa; HCs=healthy controls; SDM=seed-based d mapping;


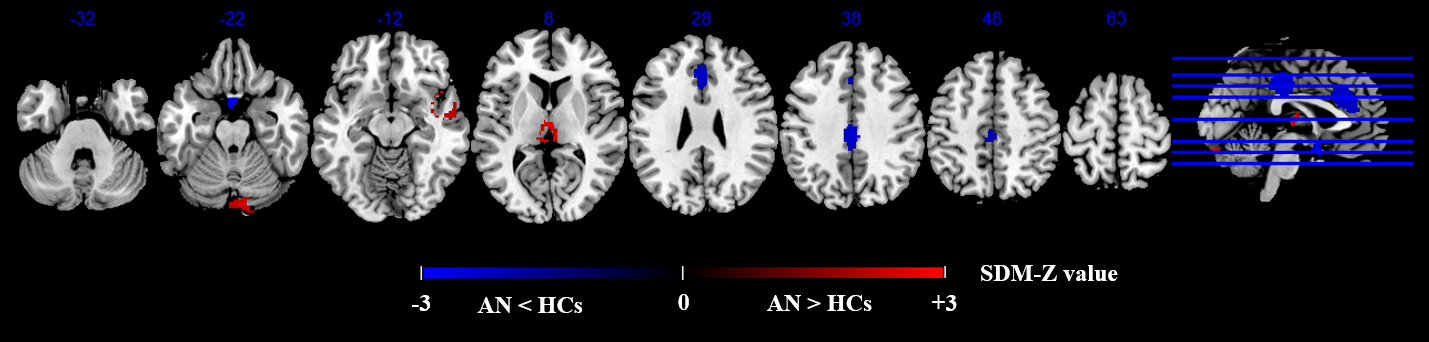


**Figure S9.** Resting-state functional activity results in AN subgroup (excluding studies with small samples [n<10]). Areas with decreased resting-state functional activity value are displayed in blue, and areas with increased resting-state functional activity value are displayed in red. The color bar indicates the maximum and minimum SDM-Z values. Abbreviations: AN=anorexia nervosa; HCs=healthy controls; SDM=seed-based d mapping;
